# Lying to your doctor: Exploring age differences and techniques to foster honest patient-doctor communication

**DOI:** 10.1177/13591053251321775

**Published:** 2025-02-24

**Authors:** Alison M O’Connor, Jessica C Frias

**Affiliations:** Mount Allison University, Canada

**Keywords:** aging, communication, health, honesty, lying

## Abstract

Honest communication between patients and doctors is essential for providing quality healthcare. The present study tested a sample of 158 younger and 149 older adults and assessed how often they lied to their doctor about their health behaviors (exercise, food/diet, alcohol use, illicit drug use, cigarette smoking). We also assessed perceptions of two communication strategies where patients reported their health concerns verbally to their doctor (direct communication) or by writing down their concerns before seeing the doctor (indirect communication). Lying to one’s doctor was common across adults, but younger adults were more likely to lie about drug use and cigarette smoking compared with older adults. Younger adults also showed a stronger preference for indirect communication and reported that this communication method would make them more honest with their doctor during a routine check-up. These results offer insight into enhancing honesty between patients and doctors.

Attending routine doctor visits (e.g. with a family doctor) is important for one’s overall physical health. For example, routinely visiting a doctor as a preventative measure can help to detect health issues in the early stages and can encourage lifestyle changes that promote more optimal health outcomes (Yom Din et al., 2014). During the visit, patients are expected to report on their health behaviors and health concerns and doctors can use such information to promote the patient’s overall health (Ha et al., 2010). Yet, the level and quality of care provided may be contingent upon the extent to which patients honestly report on their health behaviors and concerns. One of the main goals of doctor-patient communication is to facilitate the exchange of information (Ha et al., 2010); therefore, dishonesty from patients would critically interrupt this goal. While patient honesty allows the doctor to treat the patient’s concerns more effectively, there may be psychological barriers (e.g. shame; fear of stigma) that prevent honest disclosures (Frias and O’Connor, 2024; Harris and Darby, 2009; Pachankis et al., 2015; Rush et al., 2016). Shame is a negative judgment about the self and often evokes feelings of embarrassment, typically when discussing stigmatized behaviors (Tangney and Tracy, 2012); therefore, shame (and lying) can arise when discussing negative health behaviors (Harris and Darby, 2009). Patients have also been found to lie to maintain a sense of independence (e.g. concealing their participation in activities that doctors deemed unsafe; Rush et al., 2016) and to present themselves more favorably to healthcare professionals (Lapane et al., 2007).

While honesty with doctors is important for all adults, the prevalence and complexity of health concerns increases with advancing age (Cui et al., 2022; Piazza et al., 2007). Thus, it is important to study how adults communicate about their health concerns during healthcare appointments and if this differs as one ages into later life. In the present study, we assessed age differences in how often younger and older adults lied to their doctors, and we explored patient perspectives on how different doctor-patient communication scenarios may encourage honest reporting. By understanding strategies that may promote honesty in patient-doctor interactions, this can help patients feel more comfortable in healthcare settings and help doctors provide more informed and quality care for patients, and this, in turn, can advance the health of our adult population.

## Age differences in lying

Lying (i.e. intentionally providing false information to deceive or mislead others) is a common social behavior (Debey et al., 2015; DePaulo et al., 1996). Humans navigating their social world often use deceptive behaviors in various ways, including to conceal one’s wrongdoings or mistakes, to open new opportunities, to conceal one’s feelings that may cause harm or conflict with others, to alter how one is perceived by others, or simply to make things easier for oneself (DePaulo et al., 1996). For example, Debey et al. (2015) asked a large range of participants (6–77 years of age) how many lies they had told in the past 24-hours. It was found that, on average, participants told 2 lies in the past 24-hours.

Yet, research suggests that the prevalence of lying in social (Debey et al., 2015; O’Connor et al., 2022; Serota et al., 2010) and some health contexts (Frias and O’Connor, 2024; O’Connor and Evans, 2022) decreases with advancing age. For example, O’Connor and Evans (2022) studied participants aged 20–82 and found that with advancing age, adults were more honest about COVID-19 health behaviors. It is possible that greater health vulnerability in later life prompts older adults to take health concerns more seriously which, in turn, encourages honest communication. Yet, Rush et al. (2016) conducted a small case study on older adults after hospital admission, finding that older adults sometimes concealed health-related behaviors from their loved ones and doctors (e.g. lying to conceal pain and activities that they were told not to engage in). Thus, health-related dishonesty may indeed still be present in later life, though some research suggests that it may occur less frequently than in younger stages of adulthood. In younger adulthood, where future time is expansive (Carstensen et al., 1999) and where risky health behaviors are common (e.g. binge drinking, sexual experimentation, drug use), younger adults may conceal such behaviors to avoid stigmatization. Younger adults are often also more concerned with impression management and are still discovering and settling into their own identity (Arnett, 2007); therefore, they may be more apt to lie to conceal negative behaviors or to try and shape how they are perceived by others. This age-related shift in lying can also be explained through socioemotional selectivity theory (Carstensen et al., 1999). This theory posits that as one perceives that their time left to live starts to narrow, this results in a prioritization of emotionally meaningful goals where older adults are particularly motivated to feel emotionally close to others (Carstensen et al., 1999). As telling lies can instill negative feelings such as guilt, and dishonest interactions are rated as less meaningful than honest interactions (DePaulo et al., 1996), older adults may be motivated to avoid lying to maintain emotionally meaningful social interactions.

Thus, current research suggests an age-related decrease in lying into later life, and while this may transcend to health contexts, lying to doctors and spouses about health behaviors and physical recovery has been found among older adults (Rush et al., 2016), and we need more research to better understand potential age differences in lying in various health settings. Despite the growing research on age differences in lying, limited research has studied age differences in health-related lies told during doctor-patient interactions; therefore, further research is needed to better understand age differences in lying to doctors.

## Strategies to promote honesty

Given the common nature of lying in social and health contexts, yet the importance of patient honesty, it is critical to study techniques that may promote honest communication in healthcare settings to enhance quality healthcare and the overall health of our adult population. One aspect that is particularly important is the quality of doctors’ communication. Ha et al. (2010) discussed the mismatch between doctors and patients wherein most doctors perceive themselves to provide effective communication while patients commonly report dissatisfaction with their doctor’s communication efforts. Such dissatisfaction may lead to greater fear and avoidance of healthcare resources and may prevent patients from honestly disclosing sensitive health information. Indeed, Harris and Darby (2009) found that about 50% of participants reported that a doctor had said something during an appointment that made them feel ashamed, and 16% of these participants reported lying to their doctor afterward to avoid feeling ashamed or embarrassed again. Given that what doctors say and how they say it can affect patients’ experiences, it is important to explore patient perspectives of various communication methods. In the present study, we explored patients’ perspectives of more direct (verbal) and indirect (written) forms of communication between doctors and patients.

Considering that patients often report feeling shame when verbally interacting with doctors (Harris and Darby, 2009), we focused specifically on communication methods that may reduce this shame. Shame can encourage secrecy and make self-disclosure challenging (Tangney and Tracy, 2012) and has been commonly reported in verbal interactions with doctors (Harris and Darby, 2009). Therefore, it is possible that *verbally* disclosing health information when face-to-face with one’s doctor is an efficient, but shame-inducing technique that makes patients feel more vulnerable and thus reluctant to disclose. Indeed, Horan (2016) found that individuals who were less comfortable verbally discussing sex topics were more likely to conceal past sex behaviors from their partner. Thus, it is possible that verbal disclosure of health information may be challenging for some patients, warranting an investigation into other communication methods.

We explored if using more indirect communication methods, where patients could write down their concerns, would be preferred among participants and encourage honest reporting. It is possible that creating such distance offers greater psychological security when disclosing sensitive health information that may bring forth feelings of shame and embarrassment. The benefits of written disclosure (when reflecting on sensitive or traumatic information) are well documented, demonstrating that writing down one’s negative experiences or feelings can help to enhance mood and reduce psychological symptoms (e.g. depression), and this is a tool often used in therapy (e.g. Sloan and Marx, 2004). Considering the psychological benefit of written disclosure, the writing down of one’s health concerns or negative health behaviors may also be a promising avenue for difficult disclosure of symptoms or negative behaviors to doctors.

However, whether patients may be *more honest* during written or verbal communication with doctors remains unclear. Some past research suggests that adults may be more prone to dishonesty in written or online (indirect) communication settings. For example, Cohn et al. (2022) found that adults were more likely to cheat in a game when interacting with a machine compared to a human. Similarly, Nieken and Walther (2024) found that adults were more likely to choose to send a dishonest message via text rather than over a video call, suggesting that these more indirect communication methods without human interaction may make it easier to deceive. Yet, on the other hand, research on daily lying patterns finds that adolescents and adults tend to tell more lies in person than through technological or written modes of communication (DePaulo et al., 1996; Levine et al., 2013), and no research, to our knowledge, has specifically explored dishonesty across written versus verbal mediums in healthcare settings. Given evidence that lying commonly occurs in verbal interactions, and that patients report shame when verbally interacting with doctors, it is important to explore if written modes of communication may help to encourage honest reporting of health information specifically, as this psychological distance from the doctor may help to reduce shame.

## The present study

We examined how often younger and older adults reported lying to their doctors about routine health behaviors (exercise, diet, alcohol use, drug use, cigarette use). We also provided participants with two doctor-patient communication scenarios (direct verbal communication and indirect written communication) and examined which communication strategy participants preferred, which scenario would make them more honest with their doctor, and if this differed across younger and older adults.

Given that past research has found older adults to be more honest in social (Debey et al., 2015; O’Connor et al., 2022) and health (O’Connor and Evans, 2022) contexts, we predicted that older adults would be less likely to lie about all health topics relative to younger adults (H1). We also predicted that younger adults would be more likely to prefer the indirect communication method relative to older adults (H2) and that they would be more likely to report being more honest in the indirect communication method relative to older adults (H3). As younger adults have less social experience, typically less exposure to healthcare settings, and report greater shame and fear of judgment during health conversations (Frias and O’Connor, 2024) we expected that a more distant form of communication may help them to feel more comfortable in healthcare settings. Older adults, on the other hand, may view verbal communication as a more efficient communication method, and their greater experience with healthcare and lower levels of health-related shame may encourage honesty without the need for indirect communication methods.

## Method

### Participants

A total of 310 participants completed the present study; however, three participants were excluded for not meeting our age group criteria. Therefore, the present analyses were conducted on 307 participants with 158 younger adults (mean age = 25.46, SD = 3.34, and range = 18 to 35 years; 48% female) and 149 older adults (mean age = 70.22, SD = 5.14, and range = 60 to 93 years; 50% female). All participants were recruited and tested on the Prolific testing platform for convenience and were American citizens.

Among younger adults, approximately 58% of participants identified as White, 13% Black or African American, 13% Latin American, 10% East Asian, 6% Southeast Asian, 4% South Asian, and 2% or less of participants were each Indigenous or West African. Regarding the highest level of education achieved, 13% completed a post-graduate or professional degree, 49% of younger adults completed college or university, 11% were current undergraduate students, 22% completed high school, and less than 1% completed less than high school.

Among older adults, 93% of participants identified as White, 4% Black or African American, 1% Latin-American, and 1% or less of participants were East Asian. Approximately 28% of older adults completed a post-graduate or professional degree, 40% completed college or university, 1% were current undergraduate students, and 26% completed high school as their highest level of education.

### Measures

#### Health lies questionnaire

Participants reported how often they lied about their lifestyle/health behaviors to their doctor within the past year; standard questions that are asked across most doctor visits were included. Specifically, participants reported how often they lie to their doctor about (1) exercise/physical activity, (2) diet/eating habits, (3) alcohol consumption, (4) recreational drug consumption, and (5) cigarette use. Participants responded to the 5 items on a 5-point scale from 0 (*never*) to 4 (*always*). If participants did not engage in a behavior and therefore would have nothing to lie about (e.g. never smoked cigarettes), they selected “not applicable,” and this was coded as missing data.

#### Perceptions of doctor-patient communication strategies

Participants read two doctor-patient scenarios that differed in the type of communication between patients and their doctors during a routine check-up. Participants were told to imagine that they were at a routine doctor’s visit (e.g. for a physical). The first scenario (Scenario A) depicted a traditional doctor visit involving *direct communication* where the doctor verbally asked the patient questions about their general health and asked the patient if there were any concerns they wanted to talk about. Thus, this scenario involved direct verbal communication between the doctor and patient and relied on the patient to verbally state their concerns with the doctor to initiate a conversation about such concerns. The second scenario (Scenario B) depicted a form of *indirect communication* where, before seeing the doctor, the patient completed a survey about their general health and wrote down, in the survey, any specific health concerns that they wanted to discuss with the doctor. During the appointment, the doctor had the identified list of concerns from the patient and the doctor then asked the patient about each of the concerns. Thus, this scenario involved an initial phase with indirect communication where patients wrote down their concerns, followed by direct communication where, this time, the doctor initiated the conversation about such concerns.

After reading about both scenarios, participants were asked (1) which scenario they would prefer (scenario A or scenario B) and (2) which scenario would make them more honest with their doctor (scenario A, scenario B, or they would feel the same in both scenarios). Participants also reported which method their doctor currently uses (scenario A; scenario B; or neither/unsure). Of note, due to a survey error, this final question (about which method their doctor currently uses) was not included for the first 189 participants; therefore, responses to this final question were collected from only a subset of participants (*N* = 118; 50 younger adults; 68 older adults).

### Procedure

Participants completed the study online through the Prolific testing platform. Informed written consent was obtained prior to participating in the study. Participants first read a paragraph introducing the topic of lying and health to try and neutralize these topics (see online Supplemental Materials), followed by the questionnaire on how often they tell various health lies to their doctor. Participants then indicated their preference between the two doctor-patient communication scenarios. Lastly, participants provided basic demographic information, responded to questions about their health status and frequency of doctor visits, and were debriefed about the common nature of lying. This study was approved by the Research Ethics Board of Mount Allison University. Participants were compensated with £1.65 for their participation. This study was part of a larger multi-purpose study about health-related communication; therefore, participants also completed additional questionnaires, but they were not of interest for the present investigation. The questionnaires used for the present study are available in online Supplemental Materials.

### Analytic plan

The primary goals of this study were to examine age differences in health lies told to doctors and age differences in perspectives on doctor-patient communication strategies. The lie frequency data was dichotomized such that anyone who reported not telling a given lie in the past year was coded as 0 (did not lie) and anyone who did report telling the lie in the past year was coded as 1 (lied). To examine age differences in the likelihood of lying about health topics, a series of chi-squares were conducted with age group (younger vs older adults) predicting the likelihood of lying about each topic (did not lie vs lied about: exercise, diet, alcohol use, drug use, cigarette use). Participants who selected “not applicable” to a given lie topic were not included in that analysis. Binary and multinomial logistic regressions were used to test younger and older adults’ preference for direct (scenario A) or indirect (scenario B) communication scenarios, and their indication of which form of communication would make them more honest (scenario A; scenario B; or similar honesty expected across both scenarios). All data for the present study are available upon reasonable request to the corresponding author.

## Results

### Descriptive statistics

The majority of younger adults (72%) reported their physical health as good or very good and 19% of younger adults reported having a chronic health condition. Thirteen percent of younger adults said that they never visit a doctor in a typical year, 63% typically go to a doctor 1–2 times per year, 20% typically go to a doctor 3–9 times per year, and less than 1% attended doctor visits monthly or multiple times per month. The majority of older adults (87%) reported their health as fair, good, or very good, and 58% of older adults reported having a chronic health condition. Only 5% of older adults said that they never visit a doctor in a typical year, 49% typically go to a doctor 1–2 times per year, 43% typically go to a doctor 3–9 times per year, and 3% typically attended doctor visits monthly or multiple times per month.

### Frequency of lying to doctors

**Figure 1.**
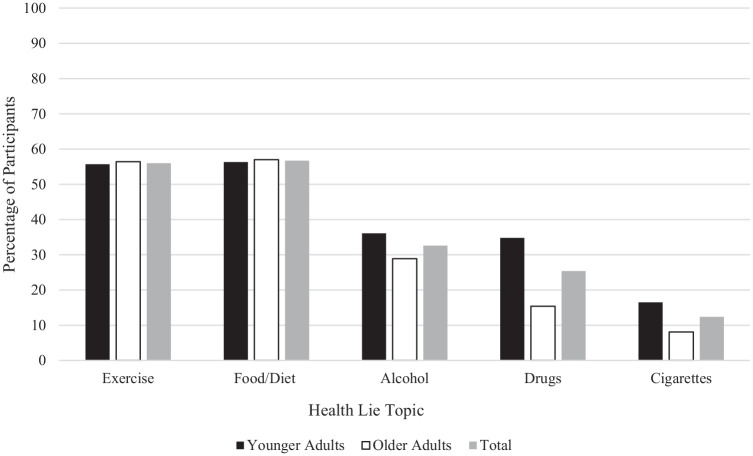
The percentage of participants who lied to their doctor across topics. Scores in this figure depict the percentage of participants who lied about each health topic to their doctor at least once in the past year.

A series of chi-square analyses were conducted to explore if age group (0 = younger adults; 1 = older adults) predicted the likelihood of lying about the various health lie topics in the past year (0 = did not report lying; 1 = reported lying). Younger and older adults did not significantly differ in the likelihood of lying about exercise (*p* = 0.895), diet (*p* = 0.809), or alcohol use (*p* = 0.232). However, relative to older adults, younger adults were significantly more likely to lie about drug use, χ^2^ (1, 281) = 18.35, *p* < 0.001, Cramer’s *V* = 0.256, and cigarette use, χ^2^ (1, 244) = 7.11, *p* = 0.008, Cramer’s *V* = 0.171. This data partially supports H1, while younger adults were more likely to lie about some health topics (supporting our prediction), there were no age differences found across several topics (contrasting our prediction). The percentage of younger and older adults who reported lying about each topic is available in Figure 1. As can also be seen in Figure 1, lies about exercise and food/diet were descriptively the most common across participants, followed by alcohol and drug use, and cigarette use was lied about the least.

### Evaluation of doctor-patient communication scenarios

When asked which communication method their doctor currently uses, 52% reported that their doctor uses direct verbal communication (Scenario A), 33% reported indirect written communication (Scenario B), and 15% reported that they were either unsure or that their doctor used neither of these methods. Thus, both communication methods presented are actively used with patients.

Overall, 60% of participants indicated that they preferred scenario B with indirect written communication over scenario A with direct verbal communication. A binary logistic regression was conducted with age group (0 = younger adults; 1 = older adults) entered as a predictor of scenario preference (0 = direct communication, 1 = indirect communication). There was a significant effect of age group on scenario preference, χ^2^ = 7.83, *p* = 0.005, *Wald*(1) = 7.71, *B* = −0.668, odds ratio = 1.95, such that younger adults were significantly more likely to prefer the indirect communication scenario (67%) compared with older adults (53%), supporting H2.

**Figure 2.**
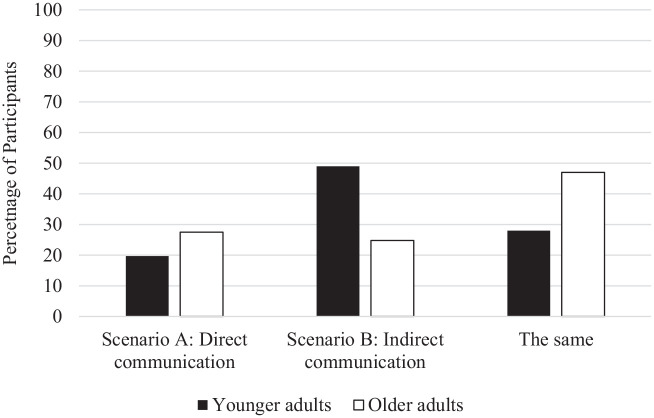
Percentage of participants selecting that Scenario A or B would encourage honesty.

Overall, 37% of participants indicated that they would be more honest with indirect communication (Scenario B), 24% indicated that they would be more honest with direct communication (Scenario A), and 37% reported that they would feel the same in both scenarios. To examine if preferences differed across age group, a multinominal logistic regression was conducted with age group (0 = younger adults; 1 = older adults) entered as a predictor of perceived honesty (more honest with direct communication; more honest with indirect communication; they would feel the same in both scenarios). Scenario B (indirect communication) was selected as the reference group. There was a significant effect of age group, χ^2^ = 21.67, *p* < 0.001, on perceived honesty across scenarios. Compared with older adults, younger adults were significantly more likely to select that they would feel more honest in the indirect communication scenario (B) over the direct communication scenario (A), *Wald*(1) = 10.61, *p* = 0.001, *B* = 1.01, odds ratio = 2.76, supporting H3. Compared with younger adults, older adults were significantly more likely to select that they would feel the same in both scenarios instead of preferring indirect communication (Scenario B), *Wald*(1) = 18.61, *p* < 0.001, odds ratio = 3.31. Thus, as evident in Figure 2, the indirect written communication strategy may be particularly advantageous in encouraging honesty among younger adults.

## Discussion

Honestly discussing one’s health behaviors is foundational for quality health care. Yet, the present study found that adults commonly lied to their doctors about health behaviors, and the most common lies told were about one’s exercise, food/diet habits, and alcohol use, and these lies were told at similar rates across younger and older adults, refuting our hypothesis (H1). However, in partial support of our hypothesis (H1), younger adults were more likely to lie to their doctor about drug and cigarette use compared with older adults. We also found that, as expected, younger adults showed a greater preference for indirect written communication with doctors (H2), and they were more likely to report being more honest with this communication strategy (H3) relative to older adults.

### Age differences in lying

The present study found that age differences in lying to doctors differed across health topics. On one hand, we found that younger adults were more likely to lie about drug use and cigarette smoking compared with older adults. This aligns with past deception research demonstrating a general age-related decline in lie-telling (Debey et al., 2015; O’Connor et al., 2022; O’Connor and Evans, 2022; Serota et al., 2010). Considering the initiation and use of drugs tend to occur in emerging and early adulthood (Palamar et al., 2013), young adults may be reluctant to disclose this information to others, including health professionals, out of fear of punishment, stigmatization, or embarrassment. Given that there are stereotypes of young adults being deviant and risk-takers (Defoe et al., 2022), this may foster greater secrecy from young adults when asked about these behaviors to distance themselves from this stereotype. Younger adults are also still exploring their identity and are more concerned with impression management (Arnett, 2007); therefore, they may be more motivated to use deceptive means to alter how they are perceived by others. It is interesting that younger adults were also more likely to lie about cigarette use compared with older adults. This may be attributed to the increase in societal stigma toward cigarette use and cigarette users that young adults would have grown up in (Palamar et al., 2013), whereas older adults would have had longer exposure to greater societal acceptance of cigarette use. Thus, again, it is possible that younger adults feel greater shame and/or embarrassment associated with their cigarette use and therefore lie about this more often to doctors compared with older adults. It is also possible that older adults are more honest about these behaviors because of their greater health vulnerability. Indeed, according to socioemotional selectivity theory (Carstensen et al., 1999), with advancing age, we tend to view our time left to live as more limited, and this can result in a reprioritization of social goals. Older adults may be experiencing this shrinking time horizon and be more cognizant of their health vulnerability, and this may encourage more honest reporting with doctors to ensure that they receive the most informed healthcare. Another consideration is the cognitive demands of lying. Research has demonstrated that lying is a cognitively taxing act that requires the effective use of various executive functioning skills (e.g. Vrij et al., 2008). As executive functions decline into later adulthood (Craik and Bialystok, 2006), it is possible that this also reduces the frequency of telling lies given the tax on cognitive resources. It can be a cognitive challenge to tell successful lies and maintain these lies over time; therefore, the decline in lying to doctors could also be, in part, attributed to older adults’ declining executive functioning skills where it is cognitively easier to tell the truth, and this honesty can also help to meet their social and health goals.

On the other hand, however, we found several topics that showed no significant age effects, demonstrating that younger and older adults lied about their exercise, food/diet, and alcohol use at similar rates. This contrasted our hypothesis and some previous research finding lower rates of health-related lying among older adults (e.g. O’Connor and Evans, 2022), but supports research demonstrating that health lies are still told by older adults (Rush et al., 2016). Previous research has demonstrated that discussing weight-related topics with doctors can be particularly shameful for patients (Harris and Darby, 2009). Such frequent lying about one’s weight-related behaviors (exercise and diet) across both younger and older adults may be associated with weight stigma (societal reinforcement of a thin ideal and discrimination against those in larger bodies; Talumaa et al., 2022). Data suggests that physicians have been reported as a primary source of weight stigma (Puhl and Brownell, 2006), and this may prevent patients from honestly reporting their exercise and diet habits to avoid being judged or stigmatized by their doctor. Indeed, over 50% of younger and older adults reported lying to their doctor about exercise and food habits. This aligns with past research demonstrating that weight stigma contributes to less patient-centered positive communication, greater patient avoidance, and overall poorer healthcare quality (Talumaa et al., 2022). Thus, the present data suggests that we may need to continue to reduce weight stigma in healthcare settings to promote honest and open communication from patients of all ages about their weight-related activities. We also found that younger and older adults reported lying about alcohol use at similar rates, presenting a different pattern than the other substances (drugs and cigarettes). This finding was unexpected and may suggest that patients, across ages, feel shame, or stigma associated with reporting alcohol use. It is possible that patients perceive their alcohol use to be less serious than other substance use, thus permitting lying at greater ease. Future research would benefit from including a measure of alcohol intake and perception of this intake along with a measure of how often this is concealed from doctors.

### Communication strategies to encourage honesty

We also explored patients’ perspectives of different communication strategies that can be used by doctors during general check-ups to explore if patients may feel more comfortable and honest with certain communication techniques. Results indicated that the indirect communication method may be particularly advantageous to use with younger adult patients as they preferred the use of indirect communication and reported that this would make them more honest with their doctor. As younger adults feel more shame surrounding their health (Frias and O’Connor, 2024) and may engage in more taboo health behaviors (e.g. sexual experimentation, substance use), using indirect communication methods may help to reduce feelings of shame and vulnerability that can accompany verbal disclosures. Thus, these results offer a potentially simple strategy to encourage honesty with younger patients (i.e. asking them to write down their concerns before seeing the doctor and allowing the doctor to initiate the verbal conversation about these concerns).

The fact that younger adults reported that they would be more honest in the indirect communication scenario compared to the direct (all verbal) communication scenario contrasts some previous research suggesting that we may be *more* deceptive in interactions without a human (Cohn et al., 2022; Nieken and Walther, 2024). It is possible these patterns differ when discussing sensitive or embarrassing health information, where participants are motivated to disclose this (for their health) but may face a psychological barrier (such as shame). The written aspect and being able to do this alone may ease reporting and make patients feel more comfortable disclosing relative to the verbal interaction with a healthcare professional. The indirect communication method may ease the reporting of small or potentially embarrassing concerns that patients may not feel comfortable voicing directly with the doctor, but it is unclear how this may present in more specialized appointments. It is also essential to consider that we assessed *perceptions* of one’s honesty in a hypothetical scenario and we did not measure actual patient honesty across these scenarios. An important next step is to test the effectiveness of these communication strategies in real healthcare settings (e.g. assess patients’ perspectives after their doctor uses the indirect vs direct communication method). If results are similar, and patients show preference for and ease of disclosure through indirect methods, then healthcare professionals may benefit from implementing this strategy into their practice.

It is important to note that although younger adults showed a *greater* preference for indirect communication relative to older adults, a majority of older adults (53%) still said that they would prefer indirect communication. Thus, older adults’ honesty may be less influenced by these communication strategies (Figure 2), but indirect communication may still be a valuable tool to use with older populations. It is possible that doctors could vary their approach based on individual needs (contributing to a patient-centered approach) as the present data suggests variability in communication preferences.

### Strengths and limitations

While we assessed common health behaviors that are ubiquitously asked across doctor visits, conversations with doctors surpass these questions and future research exploring lying to doctors about more serious health behaviors (e.g. medication use, medical history) will be important to explore. There may be other health lies that are told more frequently that were not assessed in this study. We did not aim to test a high-risk sample or a sample of adults currently undergoing medical treatments, and this survey was only presented in English; therefore, further research can be conducted to expand the generalizability of results. We also only tested younger and older adults, so we cannot draw conclusions on health-related lying throughout middle adulthood, and this would be an interesting next step for this research. Although we uncovered a potentially effective communication strategy to encourage honesty with younger patients, our results reflect patients’ perspectives on how this communication strategy would impact their interaction with their doctor and cannot address how this informs patient interactions in practice. Nevertheless, these results provide initial insight into a technique that may be effective and highlight how our preference for doctor communication methods may differ based on patient age. Considering that lying to doctors was common among younger and older adults, improvement can still be made to enhance honest and open communication between patients and doctors.

## Conclusion

The present results provide evidence that patients commonly lied to doctors about health behaviors during routine check-ups, though some of these lies were more common among younger adults. The majority of younger and older adults reported preferring indirect (written) communication with doctors, but younger adults showed greater preference for this and reported that this communication technique would make them more honest with their doctor. As lying may impede informed and quality healthcare, understanding how to foster honest patient communication is essential.

## Supplemental Material

sj-docx-1-hpq-10.1177_13591053251321775 – Supplemental material for Lying to your doctor: Exploring age differences and techniques to foster honest patient-doctor communication Supplemental material, sj-docx-1-hpq-10.1177_13591053251321775 for Lying to your doctor: Exploring age differences and techniques to foster honest patient-doctor communication by Alison M O’Connor and Jessica C Frias in Journal of Health Psychology
