# Supplementary material for: Lying to your doctor: Exploring age differences and techniques to foster honest patient-doctor communication
Source: J Health Psychol. 2025 Feb 24;30(12):3628–40. doi: 10.1177/13591053251321775 (PMC12534874; doi:10.1177/13591053251321775)
Supplement: sj-docx-1-hpq-10.1177_13591053251321775 – Supplemental material for Lying to your doctor: Exploring age differences and techniques to foster honest patient-doctor communication [file sj-docx-1-hpq-10.1177_13591053251321775.docx]

**Supplemental Material**

**Introduction provided to participants before completing the survey:**

Thank you for agreeing to participate in this study. On the next page, you will be asked about how often you lie/conceal health-related information. We lie when we say something that we know is false and we do this on purpose. Although lying is often perceived to be a negative behavior, decades of research has found that lying is extremely common. Adults tend to tell *at least* one lie each and every day. In my research, I study lie-telling, and I approach this topic from a neutral perspective (we all tell lies, and that is okay). I want to assure you that there is no judgment in this study over the lies that people tell. Lying is a normal part of our social world. To maximize the benefit of this research, I encourage you to think about your own lying and report this in the survey so that we can better understand people’s experiences. We don’t judge people for telling lies, we just want to better understand people’s lies.

**Doctor Lies Questionnaire:**

1. How often do you lie about the following lifestyle behaviors **to a doctor or a healthcare provider:**

***I lie to my doctor about...***

**How often I exercise or am physically active**

*Never*  *Rarely*  *Sometimes*  *Often*  *Always*

**How much alcohol I consume**

*Never*  *Rarely*  *Sometimes*  *Often*  *Always*

*Not applicable: I don’t drink alcohol*

**How much I use recreational (i.e., non-medical) drugs**

*Never*  *Rarely*  *Sometimes*  *Often*  *Always*

*Not applicable: I don’t do any recreational drugs*

**How often I smoke cigarettes**

*Never*  *Rarely*  *Sometimes*  *Often*  *Always*

*Not applicable: I don’t smoke cigarettes*

**What types of food I eat (my diet)**

*Never*  *Rarely*  *Sometimes*  *Often*  *Always*

**Imagine the following two scenarios:**

**Scenario A.** You are visiting a doctor for a general check-up. When the doctor comes in, they ask you questions about your general health. They then ask you if there are any health concerns that you want to talk about. You then tell the doctor, and they talk to you about these concerns.

**Scenario B.** You are visiting a doctor for a general check-up. Before you see the doctor, you complete a survey that asks about your general health and asks you to explain the health concerns that you want to talk to the doctor about. Then, when the doctor comes into the room, they have your list of concerns and asks you about them, and they talk to you about these concerns.

1. Which scenario would you prefer?

*option A* *option B*

1. Would you be more likely to be honest with your doctor in one of these scenarios?

*I would be more honest in option A*

*I would be more honest in option B*

*I would be the same in both scenarios*

1. When you visit your own current doctor or healthcare provider, which scenario do you experience?

*Scenario A*

*Scenario B*

*Neither or I’m not sure*
